# In situ reprogramming of gut bacteria by oral delivery

**DOI:** 10.1038/s41467-020-18614-2

**Published:** 2020-10-06

**Authors:** Bryan B. Hsu, Isaac N. Plant, Lorena Lyon, Frances M. Anastassacos, Jeffrey C. Way, Pamela A. Silver

**Affiliations:** 1 Department of Biological Sciences, Virginia Tech, Blacksburg, VA 24061 USA; 2 Department of Systems Biology, Harvard Medical School, Boston, MA 02115 USA; 3 Wyss Institute for Biologically Inspired Engineering, Harvard University, Boston, MA 02115 USA; 4 Department of Biological Chemistry and Molecular Pharmacology, Harvard Medical School, Boston, MA 02115 USA; 5 Laboratory of Systems Pharmacology, Harvard Medical School, Boston, MA 02115 USA

**Keywords:** Gene delivery, Bacteriophages, Microbiome

## Abstract

Abundant links between the gut microbiota and human health indicate that modification of bacterial function could be a powerful therapeutic strategy. The inaccessibility of the gut and inter-connections between gut bacteria and the host make it difficult to precisely target bacterial functions without disrupting the microbiota and/or host physiology. Herein we describe a multidisciplinary approach to modulate the expression of a specific bacterial gene within the gut by oral administration. We demonstrate that an engineered temperate phage λ expressing a programmable dCas9 represses a targeted *E. coli* gene in the mammalian gut. To facilitate phage administration while minimizing disruption to host processes, we develop an aqueous-based encapsulation formulation with a microbiota-based release mechanism and show that it facilitates oral delivery of phage in vivo. Finally we combine these technologies and show that bacterial gene expression in the mammalian gut can be precisely modified in situ with a single oral dose.

## Abstract (web summary)

It is difficult to precisely target bacterial populations in the mammalian gut. Here the authors use encapsulated phages to deliver dCas9 to *E. coli* in the mouse gut to modulate RFP expression.

## Introduction

The gut microbiome has numerous associations with human health^1^. This bacterial community contains hundreds of densely colonizing species with a composition that varies along the gastrointestinal tract (GI), between individuals, and over time^2^. The complexity of this ecosystem makes it challenging to precisely target specific bacteria without unintended impacts to the microbiota^3^. To enable the interrogation and therapeutic modification of the interactions between microbes and the host, generalizable tools are needed, especially the ones capable of modifying specific bacterial functions while minimizing disruption to non-targeted genes, microbes, and host physiology.

The efficient modification of bacterial processes within the gut is precluded by multiple biological barriers. Oral delivery is the preferable approach but remains challenging because of the acidity and proteases in the upper GI tract^4^. Enabling the delivery of biologics by this route has historically been attractive due to convenience and improved patient compliance, but is challenging due to the degradative conditions of the upper GI tract and poor intestinal absorption^5^. For applications in the gut such as phage therapy, where lytic phages are used as an antimicrobial against an enteric pathogen, physiological modification strategies have been used, including the inhibition of gastric acidification^6^ and the buffering of gastric acidity^7,8^. Although efficacious under certain circumstances, neutralization of these natural physiological barriers can be disruptive and has been associated with increased acid secretion due to feedback mechanisms^9^, susceptibility to enteric pathogens^10^, and reduced bacterial diversity in the gut^11^. An alternative approach has been to protect the phage, or other biologics, by encapsulation. Alginate, a naturally derived polysaccharide, has been of interest, because its benign gelation conditions are generally compatible with phage stability. Studies of alginate in mixtures with a pH-sensitive polymer^12^ or a single chitosan coating^13^ have shown levels of efficacy in vitro. Furthermore, co-encapsulation of phage with calcium carbonate to neutralize acid in alginate beads has shown improved phage viability in vitro^14^ and reduction of targeted species in broiler chickens^7^. Despite this progress, the efficacy of these simple encapsulation strategies are affected by variability in meal timing, intestinal motility, and individual-specific physiological conditions^15^. As such, a reliable and effective strategy to precisely deliver phage to their site of interest remains a target of research^16^. Even if these barriers can be overcome, the nature of the microbiota itself, with a dense colonization of competing species, makes the specific and durable modification of bacteria challenging.

Phages are viewed as possible therapeutics due to their capability of targeting specific bacteria, even when the bacteria are part of complex consortia. Lytic phages, which kill their cognate bacteria during phage propagation, have been of particular interest due to the prevalence of antibiotic-resistant infections. Antibacterial specificity beyond the typical species or strain level has been developed by engineering CRISPR-Cas into phages, exemplified by systems that discriminate between virulent and avirulent species of *Escherichia coli* in a moth larvae model^17^ and *Staphylococcus aureus* in a murine skin model^18^. Although temperate phages have been of less interest therapeutically, because they do not primarily pursue lysis and can integrate themselves into the bacterial genomes as prophages, they have seen some utility in antibacterial applications after modification. In one case, a temperate phage was engineered to be lytic and showed efficacy against a *Mycobacterium abcessus* infection in a patient with cystic fibrosis^19^. In another case, λ phage was engineered with a CRISPR-Cas system to increase sensitization of *E. coli* to subsequent antibacterial treatments by lytic phage and an antibiotic in vitro^20^. Although temperate phages may have some utility as antibacterial agents on their own or as adjuvants, their specificity and lysogenic conversion of bacteria offers the potential for an alternative non-lytic strategy: introducing new genes or reprogramming endogenous gene expression in precisely targeted bacteria within their natural ecosystems such as the gut microbiota. We have previously demonstrated that bacterial virulence can be repressed in vitro and in the mammalian gut using a lysogenic phage engineered to specifically repress shigatoxin expression^21^.

**Fig. 1:**
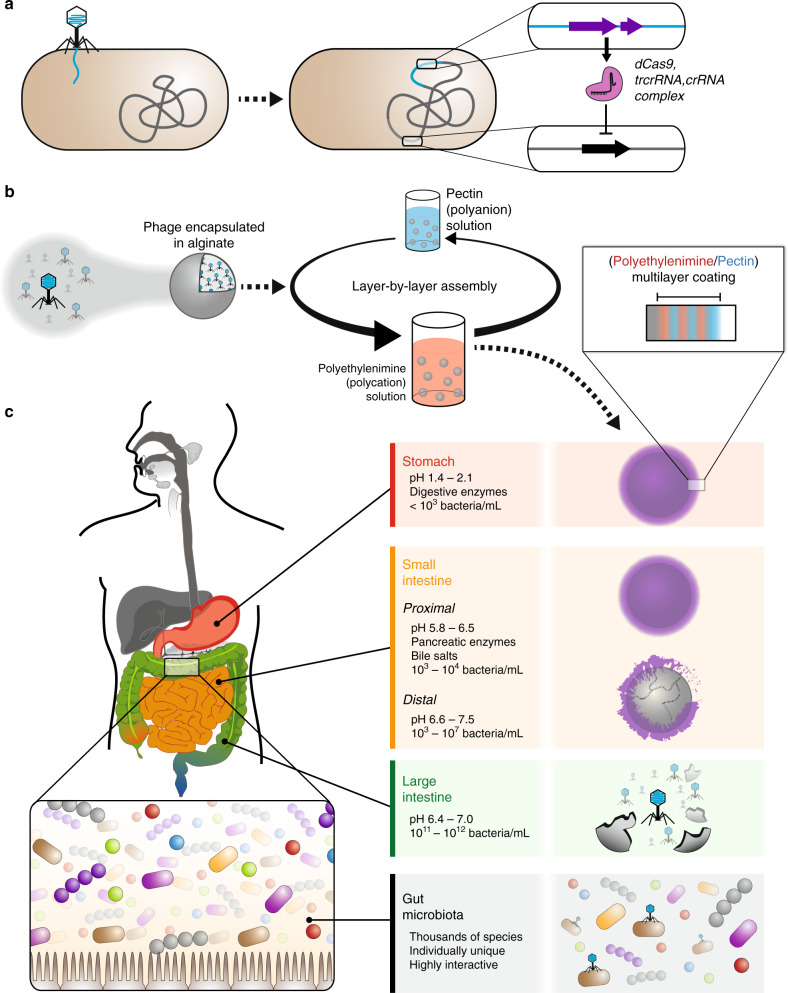
In situ modification of gut bacteria by oral delivery. Temperate phage is engineered to express dCas9, trcrRNA, and crRNA so that specific bacterial genes can be repressed (**a**). The encapsulation strategy uses calcium-mediated crosslinking of a sodium alginate solution to produce beads, which are coated in a polymeric multilayer (**b**). The digestive system has longitudinal variations in pH^27,40^ and bacteria^41^, and presents multiple challenges to biological therapeutics during their transit through the stomach, small intestine, and large intestine (**c**).

Here we report a non-invasive strategy to modify gene expression of specific bacteria in the mammalian gut via oral delivery. First, we engineer temperate phage λ to express a nuclease-deactivated Cas9 (dCas9) that specifically represses gene expression in bacteria, both in vitro and when colonizing the mouse gut (Fig. 1a). To improve survival against gastric acid and proteases, allow programmable release into the lower GI tract, and minimize potential physiological disruption to host and microbial processes, we develop an aqueous-based encapsulation formulation utilizing a layer-by-layer (LbL) assembly approach^22^ to produce a thin polymer film that protects phage during oral delivery (Fig. 1b). This biomaterial construct contains a tunable microbiota-specific release mechanism that triggers cargo release upon entry into the bacterial-dense large intestine (Fig. 1c). Finally, we demonstrate that combining these two technologies enables non-invasive and minimally disruptive in situ modification of bacteria in the mammalian gut.

## Results

### Engineered phage modifies gene expression in vitro

**Fig. 2:**
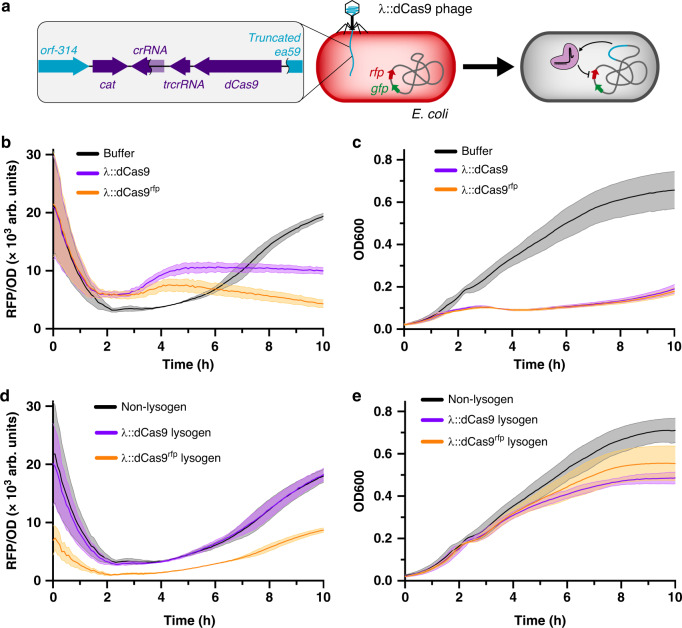
In vitro gene repression by λ::dCas9^rfp^. Scheme of in vitro experiment examining repression by engineered λ phage (**a**). *E. coli* cultures mixed with phage buffer, λ::dCas9 phage, or λ::dCas9^rfp^ phage tracked for RFP fluorescence (**b**) and bacterial density (**c**). Non-lysogenic, λ::dCas9 lysogenic, or λ::dCas9^rfp^ lysogenic *E. coli* cultures tracked for RFP fluorescence (**d**) and bacterial density (**e**). Lines represent means and shaded regions represent standard errors. Source data are provided as a Source Data file.

Engineered phage containing dCas9 represses target gene function. As shown in Fig. 2a, our engineered construct was inserted into the λ genome by replacing a portion of the non-essential b2 region^23^. We tested our system in *E. coli* containing genomically integrated *rfp* and *gfp* genes^24^, and found our plasmid-based *S. aureus* dCas9 was effective in repressing fluorescence (Supplementary Fig. 1). When phage containing crRNA targeting *rfp* (λ::dCas9^rfp^) was added to *E. coli* culture, we found that red fluorescent protein (RFP) fluorescence was markedly reduced compared to phage lacking this crRNA (λ::dCas9) (Fig. 2b). *E. coli* cultures receiving buffer showed a transitory decrease in fluorescence from ~2 to ~6 h compared to those receiving phage, which is due to an absence of initial phage propagation that occurs with the λ::dCas9 and λ::dCas9^rfp^ phage-treated samples as confirmed by the bacterial density (Fig. 2c). Furthermore, the presence of crRNA in λ::dCas9^rfp^ phage does not markedly affect bacterial growth compared to λ::dCas9 phage. To confirm gene repression is maintained once lysogeny is established, we measured RFP fluorescence from lysogens of *E. coli* and confirmed a reduced fluorescence in λ::dCas9^rfp^ lysogens compared to both non-lysogens and λ::dCas9 lysogens (Fig. 2d). Lysogeny did not affect early exponential growth but may affect bacterial density at the stationary phase (Fig. 2e).

### Engineered phage represses function of gut bacteria in situ

**Fig. 3:**
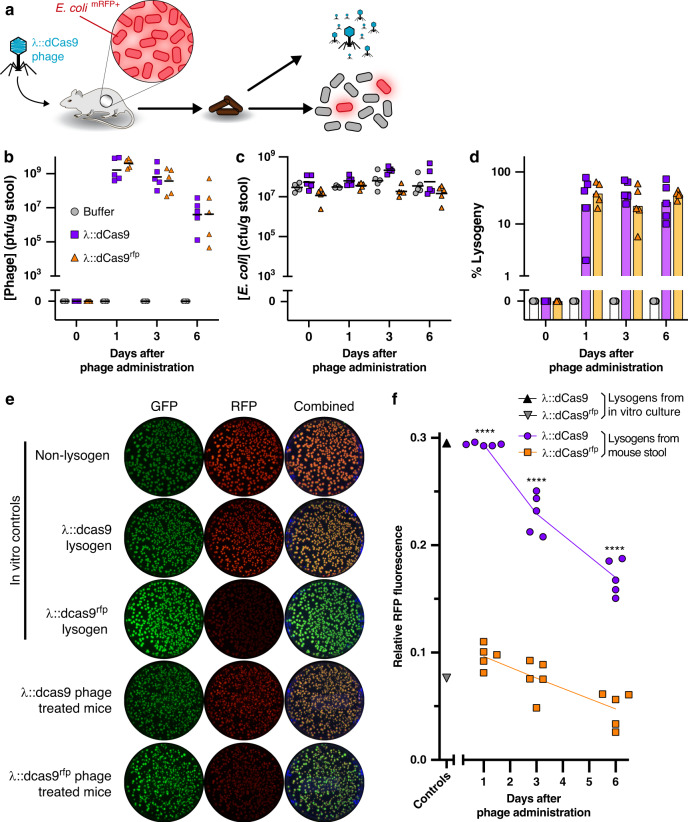
Phage delivered genetic repression in vivo. Engineered phage or vehicle was orally administered in bicarbonate solution to mice pre-colonized with *E. coli* expressing RFP and GFP (**a**). After oral administration, fecal phage (**b**), total *E. coli* (**c**), and percentage of *E. coli* lysogenized by phage were quantified (**d**). Representative fluorescence images of colonies of fecal lysogens are shown compared to in vitro-cultured controls, reproducible across three time points for each of five mice (**e**). Relative RFP intensity normalized by GFP intensity of fecal lysogens from mice. Positive and negative controls represent in vitro-cultured λ::dCas9^rfp^ and λ::dCas9 lysogens, respectively (**f**). Symbols represent individual mice (*n* = 5) with bars or lines indicating the geometric mean. Significance was calculated for preselected time-matched samples using one-way ANOVA with a Sidak post hoc test (****P < 0.0001). Source data are provided as a Source Data file.

Engineered phage lysogenizes bacteria in the mouse gut. The efficacy of our engineered phage was tested in vivo, by administration of λ::dCas9, λ::dCas9^rfp^, or vehicle (phage buffer) to mice pre-colonized with RFP-expressing *E. coli*. The fecal phage and *E. coli* concentrations were then tracked over time (Fig. 3a). To reduce degradation during gastric transit, phage solutions and vehicle were diluted tenfold into a sodium bicarbonate solution immediately prior to oral gavage. As shown in Fig. 3b, fecal phage are detectable at high concentrations soon after administration. Despite introduction of phage, total *E. coli* concentrations remain largely consistent (Fig. 3c), indicating that a moderate dose (10^7^ pfu) of temperate phage do not markedly affect concentrations of cognate bacteria in the gut. The ratio of fecal phage to fecal *E. coli* shows initially high proportions of phage that progressively decreases for both λ::dCas9- and λ::dCas9^rfp^-treated mice (Supplementary Fig. 2), which suggests an initial burst of phage amplification that reduces over time, likely due to an increasing fraction of *E. coli* lysogens. We found that a substantial fraction of fecal *E. coli* are λ::dCas9 or λ::dCas9^rfp^ lysogens soon after phage administration and remain so for the duration of our experiment (Fig. 3d).

Lysogenized *E. coli* have reduced fluorescence. We assessed functional gene repression by our engineered phage, by measuring the relative RFP fluorescence of λ::dCas9 and λ::dCas9^rfp^ lysogens isolated from mouse stool after oral phage administration. As shown in the representative culture plates in Fig. 3e, fecal *E. coli* colonies from mice receiving λ::dCas9^rfp^ phage demonstrate a maintained green fluorescent protein (GFP) and reduced RFP fluorescence compared to fecal colonies from mice receiving λ::dCas9 phage. These features were similar to in vitro-cultured λ::dCas9^rfp^ or λ::dCas9 lysogens, respectively. An ensemble view of the relative fluorescence of ~50 fecal colonies from each mouse at each time point shows that RFP repression is maintained in each mouse over time (Supplementary Fig. 3) with significant fluorescence reduction by λ::dCas9^rfp^ phage compared to λ::dCas9 phage (Fig. 3f).

### Thin-film coatings resist acid penetration

To minimize degradation of the phage when the phage is delivered orally without a buffering agent, we developed an aqueous-based encapsulation formulation capable of resisting acid penetration. Alginate beads were generated by adding an alginate solution dropwise to a stirring calcium chloride solution, which mediates gelation through ionic crosslinking. With coaxial air flow, we could tune bead size (Supplementary Fig. 4). Using an LbL assembly technique in which polyelectrolyte multilayer films are built by the alternating deposition of polycations and polyanions from aqueous solution (Fig. 1b), we coated these beads with polyethylenimine (PEI) and pectin to form thin films denoted (PEI/pectin)_*n*_ where *n* represents the number of “bilayers” deposited. On a flat substrate, these films show the characteristic exponential growth profile for weak polyelectrolytes in high-salt solutions (Supplementary Fig. 4)^25^. Pectin is included as bacteria-specific degradation mechanism to trigger bead degradation once reaching the gut. We confirmed that CaCl_2_, which is needed to ensure phage and alginate bead stability, did not disrupt polyelectrolyte complexation (Supplementary Fig. 5), and that these films remained intact in the presence of simulated gastric fluid (SGF) with pepsin (Supplementary Fig. 6).

**Fig. 4:**
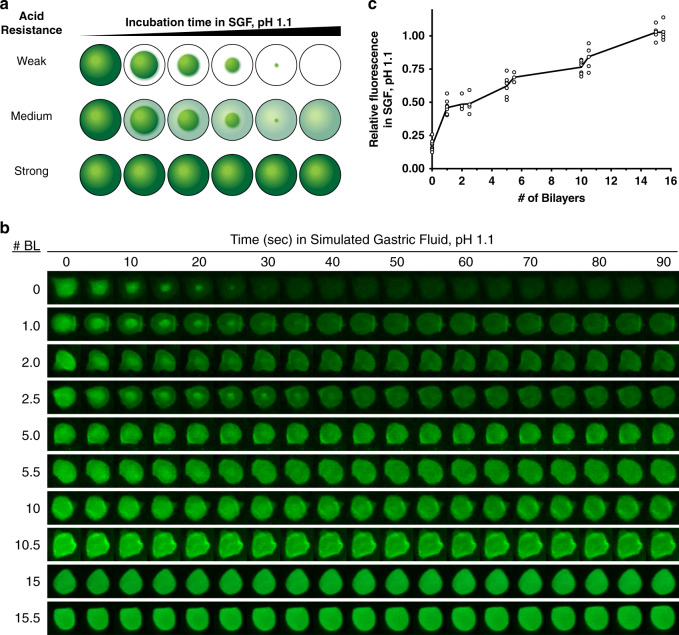
Acid resistance of alginate beads. Encapsulation of a pH-sensitive dye in the beads can gauge the pH within the beads after incubation in simulated gastric fluid (SGF) by a large or small loss in fluorescence indicating beads that have been constructed with weak, medium, or strong acid resistance (**a**). By tracking the fluorescence behavior of representative beads, non-coated (0-BL) and coated (up to 15.5-BL) alginate beads show distinct differences in acid resistance, which is repeatable across each bead analyzed in the following panel (**b**). The normalized change in fluorescence after incubation in SGF pH 1.1 for 90 s shows a relationship with the number of bilayers deposited with symbols representing individual beads (9, 8, 4, 4, 7, 3, 7, 6, 5, and 5 beads for 0, 1, 2, 2.5, 5, 5.5, 10, 10.5, 15, and 15.5 bilayers, respectively) and the line indicating the mean (**c**). Source data are provided as a Source Data file.

LbL-film-coated alginate beads are resistant to external pH changes. We quantified the acid resistance of these thin films by encapsulating an Oregon Green 488–dextran conjugate, which contains a pH-sensitive dye that fluoresces under neutral pH and quenches in acid (Supplementary Fig. 7). When suspended in SGF pH 1.1, the fluorescence quenching of these beads indicates the acidification of the bead interior from its initially physiological conditions of pH 7.5 (Fig. 4a). As shown in Fig. 4b, acid readily infiltrates the entirety of uncoated alginate beads (0-BL) within ~30 s. Increasing the number of bilayers, i.e. film thickness, increases acid resistance. Quantification of relative fluorescence intensity reveals that increasing the number of bilayers deposited increases acid resistance with 15 and 15.5-BL coatings maintaining nearly unchanged internal conditions during the first 90 s (Fig. 4c). Furthermore, the acid resistance is retained during prolonged incubation of 2 h at 37 °C (Supplementary Fig. 8). Encapsulating λ phage into alginate beads coated with (PEI/pectin)_15.5_ films revealed superior in vitro stability against acid compared to λ phage in uncoated alginate beads and non-encapsulated free λ phage (Supplementary Fig. 9). When stored at 4 °C for 8 months, phage encapsulated in these LbL-coated beads retained 31 ± 8% (mean ± SD) of initial phage viability, which is comparable to similarly stored free phage (54 ± 21%).

### Encapsulation protects phage during oral delivery

Non-encapsulated free phage is degraded when administered orally to mice. For a baseline measure of the degradative conditions of the stomach, we administered increasing concentrations of λBH1 phage in water to mice pre-colonized with *E. coli* MG1655. λBH1 phage is an engineered λ phage encoding an antibiotic resistance marker allowing us to quantify lysogens by antibiotic selection^21^. Overnight fasting can raise the gastric pH (pH ~ 4)^26^, so to preserve gastric acidity, we gavaged mice in the fed state to keep the gastric conditions (pH ~ 3)^26^, which is closer to that of humans (pH ~ 2)^27^. We found the mouse gastric pH to be 2.39 ± 0.56 (mean ± SD, *n* = 5). After administering steadily increasing doses (10^1^ pfu to 10^8^ pfu) of λ phage suspended in water, we monitored fecal phage and lysogen content in mouse stool for 3 days. As shown in Supplementary Fig. 10, no phages or lysogens were detected in the feces for doses up to 10^7^ pfu, despite the persistent colonization by *E. coli* that would readily amplify any phage surviving oral administration. It is not until a dose of 10^8^ pfu that phage and lysogens are detected in the stool. The necessity of such a high dose indicates λ phage is readily degraded when orally administered in vivo^28^.

**Fig. 5:**
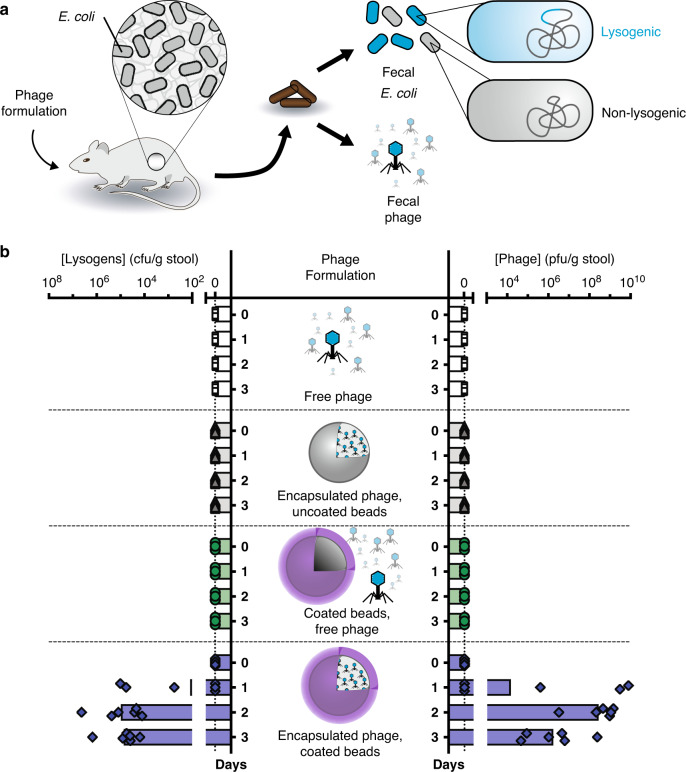
Efficacy of oral encapsulation formulation on bacteriophage survival in mice. Mice pre-colonized with *E. coli* were orally administered phage formulations (**a**). Mice were administered either free phage (*n* = 5), phage encapsulated in uncoated beads (*n* = 6), a mixture of free phage with empty Layer-by-Layer (LbL)-coated beads (*n* = 3), or phage encapsulated in LbL-coated beads (*n* = 6). “LbL-coated” indicates coatings of (polyethylenimine/pectin)_15.5_ films. Fecal concentrations of lysogens and phage were quantified over time (**b**). Symbols represent individual mice with bars indicating geometric mean. Source data are provided as a Source Data file.

LbL-coated encapsulation formulations protect phage during oral delivery in mice. To determine the protective efficacy of our encapsulation formulation during oral administration, mice were gavaged with a low dose (10^3^ pfu) of phage in various formulations suspended in water (Fig. 5a). Due to size restrictions in mouse anatomy, we used 0.65 mm diameter alginate beads (Supplementary Fig. 4). As shown in Fig. 5b, oral administration of phage encapsulated in alginate beads coated with (PEI/pectin)_15.5_ films resulted in phage detected in stool for all mice within 2 days and continued so for 2 weeks (Supplementary Fig. 11). In vitro studies indicate that phage is rapidly released from these beads (Supplementary Fig. 12). We observed an initial burst of phage relative to *E. coli* (Supplementary Fig. 13) similar to our previous experiment (Fig. 3 and Supplementary Fig. S2), except that a greater proportion of phage was reached. This is likely due to a lower initial dose (10^3^ pfu vs. 10^7^ pfu), which potentiates greater initial phage propagation. By contrast, phage alone or phage co-administered with empty alginate beads coated with (PEI/pectin)_15.5_ films did not survive oral administration (Fig. 5b) despite the presence of *E. coli* capable of phage amplification (Supplementary Fig. 14). The latter indicates that encapsulation is necessary, and that the beads do not provide protection by somehow inactivating the digestive process. Similarly, phage encapsulated in alginate beads without the LbL coating were completely inactivated, confirming that the LbL films are the essential protective element.

### Minimally disruptive modification of gut bacteria

**Fig. 6:**
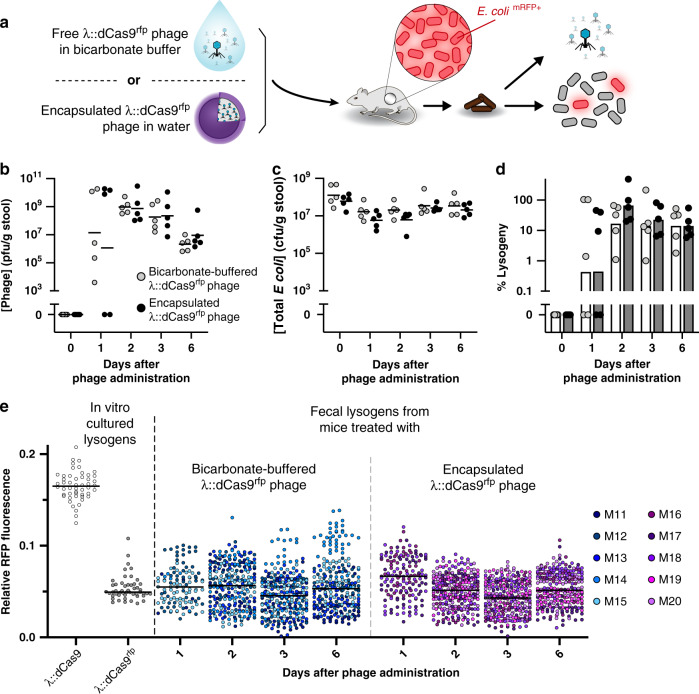
Encapsulation protects oral phage but does not impair function. Free λ::dCas9^rfp^ phage in bicarbonate buffer (to ensure survival) or encapsulated λ::dCas9^rfp^ phage in water were orally administered to mice pre-colonized with *E. coli* expressing RFP and GFP (**a**). After oral administration, fecal phage (**b**), total *E. coli* (**c**), and percentage of *E. coli* lysogenized by phage were quantified (**d**). Symbols represent individual mice (*n* = 5) with bars or lines indicating the geometric mean. Ensemble view of relative fluorescence from lysogens shown with in vitro-cultured controls (**e**). Symbols represent 50 individual colonies per mouse per sample or the maximum discernable colonies (Mouse 1, Day 1 = 5 colonies; M13, D1 = 0; M14, D1 = 8, D6 = 49; M15, D1 = 49; M19, D1 = 0; M20, D1 = 0) with lines indicating the median. Source data are provided as a Source Data file.

Encapsulated phage modifies gut bacteria in situ without compromising the gastric barrier. In Fig. 3, we demonstrate that λ::dCas9^rfp^ phage significantly represses RFP fluorescence in gut bacteria compared to λ::dCas9 phage, which lacks targeting to the *rfp* gene of *E. coli*. In Supplementary Fig. S10 and Fig. 5, we demonstrate that phage administered in water is readily inactivated during oral administration unless the stomach acid is neutralized, phage is given at very high concentration, or phage is encapsulated into our LbL-coated alginate beads. To determine whether encapsulation impairs λ::dCas9^rfp^ phage performance in situ, we administered a low concentration (5 × 10^3^ pfu) of this phage either in LbL-coated beads suspended in water or as free phage mixed with bicarbonate buffer to neutralize the stomach acid (Fig. 6a). Examination of mouse stool after oral phage administration reveals similar fecal phage concentration (Fig. 6b), total fecal *E. coli* concentration (Fig. 6c), lysogenic conversion of fecal *E. coli* (Fig. 6d), and ratio of fecal phage to *E. coli* (Supplementary Fig. 15). When examining the relative RFP fluorescence of individual λ::dCas9^rfp^ lysogen colonies, both buffered and encapsulated phage provided similar levels of RFP repression for the duration of the study (Fig. 6e).

## Discussion

In this study, we demonstrate microbiological and biomaterial advances that are individually useful and can be combined for precise targeting of bacteria in the gut. First, we describe a precise and programmable approach for the in situ modification of bacteria and bacterial transcription using temperate phage and RNA-guided dCas9. Next, we show that a microbiome degradable acid-resistant coating and encapsulation formulation releases engineered phage in the lower GI tract and maximizes the efficacy of oral delivery while minimizing potential physiological disruption. Combined, these technologies demonstrate a non-invasive, durable, and targeted approach towards modifying bacterial function in the gut.

Manipulating bacterial processes in situ could be a powerful tool for dissecting the causal relationship between microbial and host physiology. Current approaches, such as the use of small molecules to interrogate the impact of microbial metabolites, are under investigation^29^ but are limited by off-target effects^30^. Our approach allows for a fine-tuned genetic control of a single gene within a single microbe, which makes it possible to systematically screen diverse genetic targets, characterize cause–effect relationships, and identify cascading effects to the surrounding microbiota and host. As a therapeutic, our work outlines a generalizable strategy for durable modification of gut bacteria using a single, self-propagating dose.

The life cycles of temperate phage—lysogeny and lysis—are important factors in the success of our described approach. Lysogeny allows for the integration of genetic material into the bacterial genome, whereas lysis enables phage amplification to reach more bacterial hosts beyond the initial dose. Our work leverages these advantages to achieve a high rate of lysogenic conversion in the murine gut from a single relatively low dose: ~10^3^ pfu phage compared to ~10^8^ cfu/g stool of *E. coli*. We demonstrate gene repression for at least 6 days under our controlled conditions, but various stressors such as inflammation^31^ or antibiotics^32^ are known to induce prophages. This may impact the degree of lysogeny and warrants further investigation.

With this exquisite specificity comes a possibly narrow efficacy. As microbial functions can be shared across co-colonizing bacteria, multiple phages might be required to achieve the desired results. This approach is already used in traditional phage therapy, where cocktails are used to broaden the host range of targeted bacteria. Another general consideration when deploying phages is the development of resistance. Prophages are prevalent among gut bacteria^33^ and likely persist, because they confer fitness advantages to the bacterial host^34^, suggesting that engineered temperate phages could similarly be tolerated in the gut microbiota.

As we gain greater insight into the extent of host–microbiome interactions, the development of precision tools to modify the function of species within their ecosystem could improve both the specificity and durability of the intervention. Although there is great interest in the human gut, the microbiomes of other bodily regions such as the mouth, skin, bladder, nose, ears, eyes, and lungs also have therapeutic potential. In addition, microbiomes in animals, plants, insects, soils, and bodies of water may all be targets for the modification of specific microbes.

## Methods

### Molecular cloning and phage engineering

Golden Gate Assembly was used for cloning plasmids. Q5 Hot Start polymerase was used to amplify the proC promoter, λ phage homology arms, pACYCDuet origin of replication, and pACYCDuet chloramphenicol resistance cassette. Restriction sites were also added during PCR. A DNA sequence including the coding sequence of *S. aureus* dCas9, the dCas9’s tracRNA under a constitutive promoter, and the dCas9’s crRNA under a constitutive promoter, was ordered from IDT as two gBlocks. We used chloramphenicol acetyltransferase as the antibiotic resistance and Cas9 from *S. aureus*^35^ because of their minimal size. Cas9 was modified with nuclease-inactivating mutations in the HNH and RuvC catalytic regions^36^. Golden Gate reactions were run using 10× T4 Ligase Buffer (Promega), T4 Ligase (2,000,000 units/mL, NEB), and bovine serum albumin (10 mg/mL, NEB), as well as the appropriate restriction enzyme, either Eco31I, Esp3I, or SapI (Thermo FastDigest). Golden Gate reactions were desalinated using drop dialysis (for a minimum of 10 min) and electroporated in DH10β Electrocompetent Cells (Thermo Fischer). Plasmids were verified by sequencing all junctions and the entirety of the gBlocks. The gRNA spacers were synthesized as complementary oligos, then added to plasmids by Golden Gate after being annealed and phosphorylated. This was done by incubating the oligos with T4 Ligase Buffer (NEB) and T4 Poly Nucleotide Kinase (NEB), heating to boiling, and then slowly cooled to room temperature. Annealed oligos were added to plasmids using Eco31I. The gRNA spacers were verified by sequencing.

To generate a crude phage lysate containing the desired recombinant phage, cultures of log-phase *E. coli* C600 containing dCas9 plasmids grown in TNT (tryptone-NaCl-thiamine) media with 25 μg/mL chloramphenicol were pelleted and resuspended into an equal volume of TNT media. Plasmids contained 40 bp homology regions to *ea59* and *orf-314* genes, to facilitate homologous recombination of the dCas9 construct into λ phage. In a double agar layer plaque assay method^23^, 100 µL of this culture was mixed with 100 µL of serially diluted λ phage in phage buffer (50 mM Tris, 100 mM sodium chloride, 10 mM magnesium chloride, and 0.01% gelatin at pH 7.5) and then mixed with 3 mL of molten top agar (TNT with 0.3% agar) and immediately poured onto TNT agar plates to harden. After overnight culture at 37 °C, the top agar from plates containing the highest density of individual plaques were resuspended into 5 mL of phage buffer with gentle rocking at 4 °C for 2 h. These suspensions were then pelleted with the supernatant filtered through 0.45 μm syringe filters, yielding crude phage lysates.

*E. coli* C600 grown to late log in TNT with 0.4% maltose was concentrated by pelleting and resuspension to ~10^10^ cfu/mL. Two hundred microliters of this bacterial suspension was mixed with 200 µL of crude phage lysate and incubated at 37 °C for 2.5 h, statically. Cultures were then plated onto Lysogeny Broth (LB) agar with 34 μg/mL chloramphenicol and incubated overnight at 37 °C. For plaque purification, phage was isolated by culturing streak-purified colonies in TNT overnight at 37 °C, then treated with chloroform and pelleted. Plaques were then generated from serial dilutions of the supernatant by double overlay plaque assay with *E. coli* (*rfp*^*+*^, *gfp*^*+*^). After overnight incubation at 37 °C, plaque centers were picked and streaked onto LB with chloramphenicol. Resultant colonies were checked for GFP fluorescence and PCR amplicons to confirm the correct bacterial host and presence of phage, respectively.

### In vitro fluorescence measurements

In flat-bottom, 96-well fluorescence plates, 180 μL of log-phase *E. coli* (*rfp*^*+*^, *gfp*^*+*^) cultured in TNT was diluted to OD600 ~ 0.05 (1 cm pathlength) and mixed with 20 µL of λ::dCas9 phage or λ::dCas9^rfp^ phage for a final multiplicity of infection ~ 1.0, or phage buffer. Plates were shaken at 37 °C for 10 h with measurements of OD_600_, GFP fluorescence (ex 485 nm/em 528 nm), and RFP fluorescence (ex 555 nm/em 584 nm) at 5 min intervals (BioTek Synergy H1MF and BioTek Gen5 v3.03). Studies of non-lysogens, λ::dCas9 lyosgens, or λ::dCas9^rfp^ lysogens were set up similarly, except that 200 µL of log-phase cultures were used.

### Bead generation and film coating

Alginate beads were generated according to established methods^37^. A 1% sodium alginate solution in phage buffer was dissolved with stirring, heated to a brief boil, and then cooled to 4 °C. This solution was added dropwise with a 30-gauge needle (BD) at 50 mL/h via syringe pump (New Era Pump Systems) to a stirring solution of phage buffer containing 100 mM calcium chloride. After complete addition, the bead suspension was stirred for an additional hour, then washed twice with phage buffer containing 100 mM calcium chloride, and stored in the same solution at 4 °C. For encapsulation, bacteriophage was included in the sodium alginate solution prior to addition to the calcium chloride bath. Average bead diameters were determined from at least 100 beads using ImageJ.

We then coated these alginate beads in polyelectrolyte multilayer films. For one bilayer, beads were incubated for 15 min in a polycation solution and washed twice, then incubated for 15 min in a polyanion solution and washed twice. This process was repeated *n*-times for *n*-bilayers. The polycation solution consisted of 2 mg/mL branched PEI (750 kDa) and the polyanion solution consisted of 2 mg/mL apple pectin. Both polyelectrolyte solutions and wash buffer were formulated in phage buffer with 100 mM calcium chloride, pH 7.5. After film deposition, beads were stored at 4 °C.

### Film characterization

Multilayer films were assembled onto silicon wafers (University Wafer) using microfluidic devices fabricated using soft-lithography techniques^38^ and described here briefly. Masters were generated on precleaned 4 inch Si wafers by spin coating SU-8 2050 photoresist (~90 μm) and soft-baking at 65 °C for 5 min followed by 95 °C for 10 min. After film exposure using a mask and mask aligner (Karl Suss MJB3), films were baked at 65 °C for 2 min and at 95 °C for 8 min followed by development in SU-8 developer and isopropanol. The photolithographic mask used is shown in Supplementary Fig. 16. Polydimethylsiloxane (PDMS) molds were prepared by mixing curing agent with prepolymer (Down Corning Sylgard 184) at a 1 : 10 ratio and pouring onto the silanized master. After curing at 60 °C for 30 min, the PDMS mold was peeled from the master and adhered to precleaned silicon wafers for use. Multilayer films were assembled in devices by adding 10 µL of 2 mg/mL PEI solution in phage buffer for 5 min, washing twice with phage buffer, then adding 10 µL of 2 mg/mL pectin solution in phage buffer for 5 min, and washing twice with phage buffer. At the end of step, solutions were removed from wells by aspiration. Film thicknesses were measured by profilometry (KLA-Tencor).

### In vitro characterization of bead function

We assessed the internal pH of the alginate beads by incorporation of 150 µg/mL (~100 µM dye) of the polymer-dye conjugate, dextran-Oregon Green 488 (70 kDa M_W_, Life Technologies), in the sodium alginate solution prior to gelation. In 24-well plates, we resuspended the beads into 1 mL of SGF pH 1.1 and imaged the fluorescence using a macroscope device^39^. SGF consisted of 34 mM sodium chloride and 85 mM HCl pH 1.1. The relative change in fluorescence for each bead was determined by first subtracting the raw background intensity immediately adjacent to the beads from the raw intensity within the center of the beads and then taking the relative change in fluorescence as determined by the fraction of remaining fluorescence after 90 s compared to its initial fluorescence, immediately after the addition of SGF. For the qualitative comparison in change of fluorescence over time, identically adjusted the contrast and brightness for each bead montage over time was used.

We determined the in vitro protective ability of encapsulation by suspending individual beads containing 1.1 × 104 pfu/bead in 0.5 mL of SGF (pH ~ 1.1 to 7.4) for 15 min at 37 °C. Bacteriophage was released by re-suspending the beads into phage buffer containing 0.5 mg/mL alginate lyase and 5 mg/mL pectin lyase with shaking at 37 °C for 10 min with mechanical disruption using a sterile wooden stick and then continued shaking at 37 °C for an additional 10 min. This process did not affect phage titers (Supplementary Fig. 17). Free bacteriophage was diluted 1000-fold into 0.5 mL of SGF (pH ~ 1.1 to 7.4), incubated for 15 min at 37 °C, and then diluted 50-fold into phage buffer. Samples were kept on ice until used in for the plaque assays described above.

Phage release from alginate beads coated with (PEI/pectin)_15.5_ films was determined by resuspension into 1 mL of phage buffer containing 0.5 mg/mL of alginate lyase and 0.5 mg/mL of pectin lyase followed by shaking at 37 °C. The quantity of phage released into the supernatant was assayed by plaque assay, whereas the phage remaining in the beads was released by mechanical disruption followed by plaque assay.

### Animal studies

All animal work was approved by the Harvard Medical School IACUC under protocol 04966. Upon arrival, 6- to 7-week-old female BALB/c mice (Charles River) were acclimated for a week under ambient humidity, 12 h light/dark cycles, and 72 ± 1 °F prior to experiments. A solution of 5 g/L streptomycin sulfate USP grade (Goldbio) was provided in the drinking water 1 day prior to oral gavage with 100 µL of streptomycin-resistant *E. coli* MG1655 or *E. coli* (*rfp*^*+*^, *gfp*^*+*^) using 20-gauge polytetrafluoroethylene animal feeding needles (Cadence Science). The gavage solution of *E. coli* was prepared by inoculating an overnight culture (~16–20 h) in LB with 100 µg/mL streptomycin, then washing twice by pelleting and then re-suspending in an equal volume of phosphate-buffered saline (PBS), and then diluting tenfold into PBS to yield ~10^7^–10^8^ cfu/mL. One day after administration of *E. coli*, 100 µL of phage solution was administered by oral gavage.

Testing the efficacy of engineered phage for repressing RFP fluorescence (Fig. 3). Solutions of vehicle (phage buffer), either 10^9^ pfu/mL of λ::dCas9 phage or 10^9^ pfu/mL of λ::dCas9^rfp^ phage, were diluted tenfold into 0.1 M sodium bicarbonate followed by immediate administration of 100 µL to mice by oral gavage.

Testing the susceptibility of phage to inactivation during oral delivery (Supplementary Fig. 10 and Fig. 5). To test free phage, λBH1 was diluted into water and then 100 µL was administered to mice by oral gavage. Increasing doses of 10^1^, 10^3^, 10^5^, 10^7^, and 10^8^ pfu were administered at 3-day intervals. λBH1 phage or lysogens were not detected in stool until after the highest dose. To test phage susceptibility to degradation in different formulations, λBH1 phage free in solution, mixed (i.e., not encapsulated) with LbL-coated alginate beads, encapsulated into alginate beads without coating, or encapsulated into LbL-coated alginate beads at 20 pfu/bead. The LbL coating consisted of a (PEI/pectin)_15.5_ film. Phage solutions were diluted into water immediately prior to oral administration of 100 µL to mice, whereas encapsulated phage was resuspended into water immediately prior to oral administration of 100 µL containing 50 beads. All solutions and suspensions were administered using 16-gauge polyurethane feeding needles (Instech Labs) to enable administration of beads.

Testing the efficacy of encapsulated engineered phage in repressing RFP fluorescence (Fig. 6). To test the efficacy of encapsulated λ::dCas9^rfp^ phage, 100 µL of a low-dose (5 × 103 pfu) of free phage or phage encapsulated into LbL-coated beads with (PEI/Pectin)_15.5_ films at 710 pfu/bead were administered to mice by oral gavage. Free λ::dCas9^rfp^ phage was diluted tenfold into 0.1 M sodium bicarbonate immediately before administration to mice. Encapsulated phage (in seven beads) was resuspended into water immediately before administration to mice. Solutions and suspensions were administered by oral gavage using 16-gauge polyurethane feeding needles (Instech Labs) to enable administration of beads.

To quantify the colonization within the mouse gut, daily stool samples were obtained. To quantify phage, within 30 min of excretion stool was suspended into 1 mL of phage buffer using sterile hospital sticks and then kept on ice. After 10 min, a few drops of chloroform was added to kill bacteria without affecting the bacteriophage^23^. After an additional 10 min, the solution was pelleted at 2100 × *g* for 10 min and then the supernatant diluted into phage buffer and quantified by plaque assay against the indicator bacteria, *E. coli* C600 using a double agar layer approach. To quantify bacteria, stool was frozen at −80 °C within 30 min of excretion and then immediately prior to analysis, thawed at room temperature, resuspended into 1 mL of PBS with vortexing for 10 min at 4 °C, and then gently centrifuged at ~2.5 × *g* for 20 min to allow debris to settle, while leaving bacteria in suspension. *E. coli* was quantified by plating 100 μL of serial tenfold dilutions in PBS onto MacConkey agar (Remel) supplemented with (100 μg/mL) streptomycin to quantify total *E. coli* or MacConkey agar supplemented with 100 μg/mL streptomycin sulfate and 50 μg/mL kanamycin sulfate, to quantify bacteriophage lysogens. Fluorescence from *E. coli* colonies was measured by plating onto LB with 34 μg/mL chloramphenicol and incubated at 37 °C for 2 days. Fluorescence images were taken with a Bio-Rad alpha imager with Bio-Rad Image Lab v6.0 and fluorescence intensity quantified by ImageJ.

To determine the gastric pH, mice were allowed free access to food and water, then killed under CO2 and cervical dislocation, dissected, and a pH probe was immediately inserted into the gastric contents for measurement.

All data were analyzed using Microsoft Excel for Mac v16.35 and Graphpad Prism v8.4.2.

### Reporting summary

Further information on research design is available in the Nature Research Reporting Summary linked to this article.

## Supplementary information

Supplementary Information

Reporting Summary

## Data Availability

The sequences of plasmids generated in this study have been deposited in GenBank with accession codes MT882690 and MT882691. Materials are available upon request. Source data are provided with this paper.

## Source data

Source Data
