# Supplementary material for: In situ reprogramming of gut bacteria by oral delivery
Source: Nat Commun. 2020 Oct 6;11:5030. doi: 10.1038/s41467-020-18614-2 (PMC7538559; doi:10.1038/s41467-020-18614-2)
Supplement: Supplementary file 1 — Supplementary Information [file 41467_2020_18614_MOESM1_ESM.pdf]

SUPPLEMENTARY INFORMATION

In situ reprogramming of gut bacteria by oral delivery

Hsu et al.

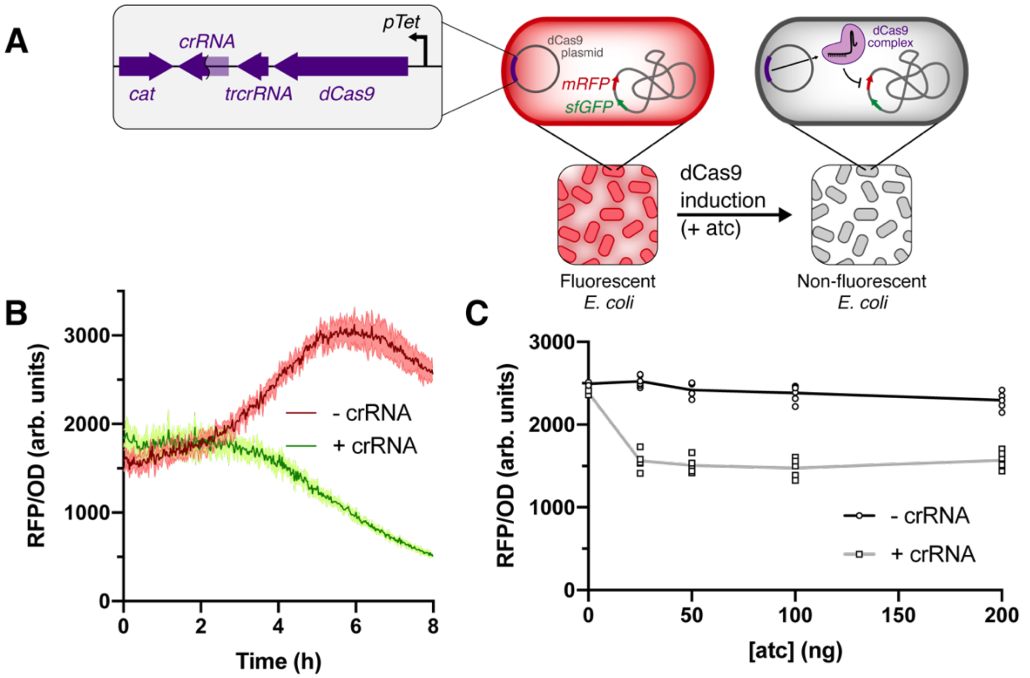

Supplementary Figure 1. **Plasmid based *in vitro* gene repression.** *E. coli* transformed with atc inducible expression of dCas9 with and without crRNA targeting *rfp* (A). Relative fluorescence of over time after induction with 25 ng atc (B) and as a function of atc concentration after 6 h incubation (C). Symbols represent independent experiments (-crRNA, 0 ng = 2, 50 ng = 4; +crRNA, 0 ng = 2, 100 ng = 5; all other samples = 6) and lines represent the mean. Source data are provided as a Source Data file.

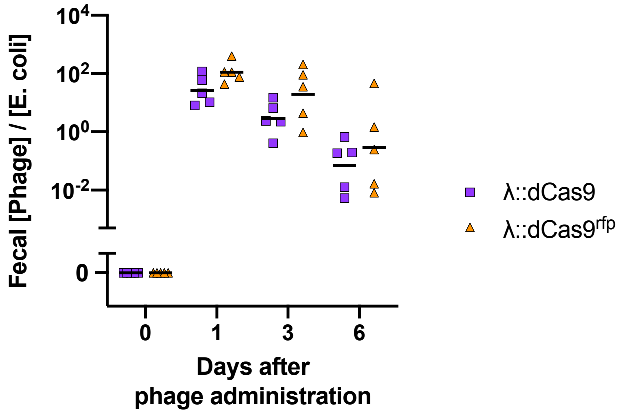

Supplementary Figure 2. **Ratio of phage to bacteria.** The concentration of relative phage to bacteria present in each fecal sample for each mouse. Symbols represent individual mice (n=5 per group) and lines represent the geometric mean. Source data are provided as a Source Data file.

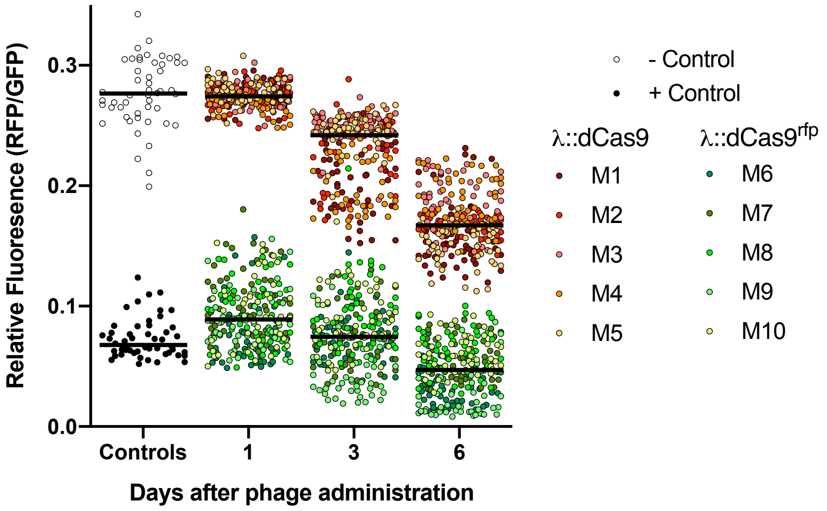

Supplementary Figure 3. **Ensemble view of relative colony fluorescence of fecal E. coli.** 50 colonies from mice receiving either λ::dCas9 (n=5) or λ::dCas9^{rfp} phage (n=5). Controls represent lysogens after in vitro culture. Lines represent the median. Source data are provided as a Source Data file.

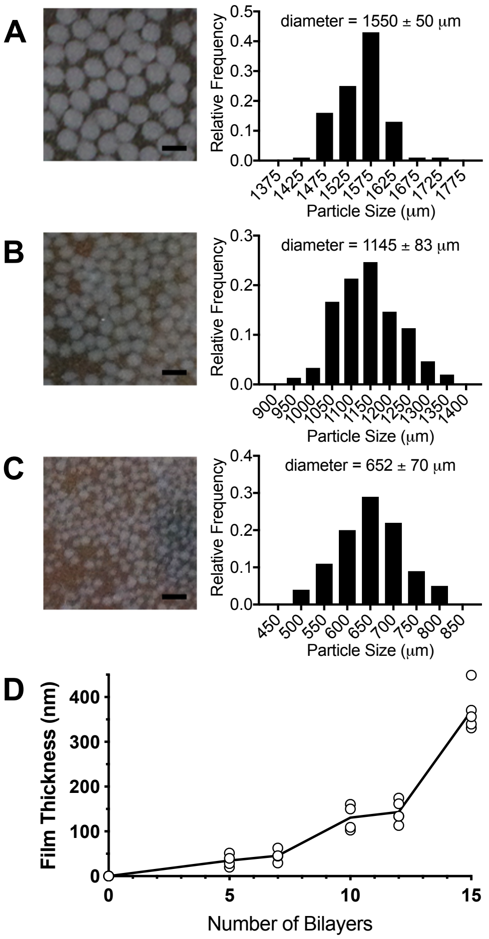

Supplementary Figure 4. **Properties of encapsulation formulation.** Alginate beads are generated by dropwise addition of an alginate solution into an aqueous calcium chloride solution, assisted by coaxial air flow at 0 standard cubic feet per minute (scfm) (A), 50 scfm (B), and 100 scfm (C). The film thickness of the subsequent multilayer thin film of (polyethylenimine/pectin)$_n$ was characterized on silicon wafers (D). Bars and line for bead diameters and film thickness, respectively, are represented as the mean. Symbols represent independent experiments (n=4 for 5, 7, and 10 bilayers; n=5 for 12 and 15 bilayers). Scale bars = 2 mm. Source data are provided as a Source Data file.

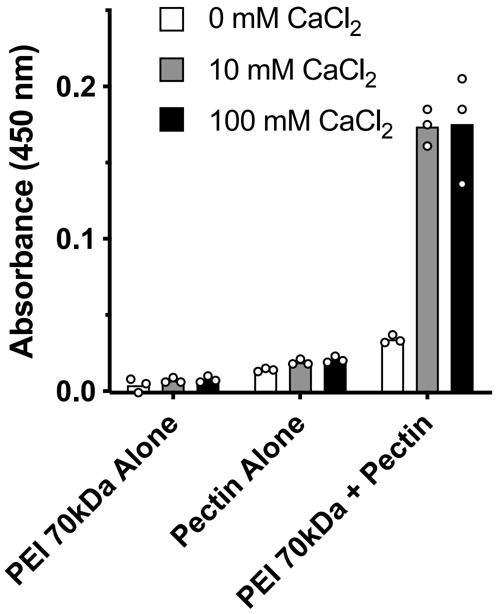

Supplementary Figure 5. **Polyelectrolyte complexation in calcium chloride.** Absorbances at 450 nm of polyelectrolyte complexes between polyethylenimine (PEI) and pectin in the presence of calcium chloride. Symbols represent independent experiments (n=3) and bars represent the mean. Source data are provided as a Source Data file.

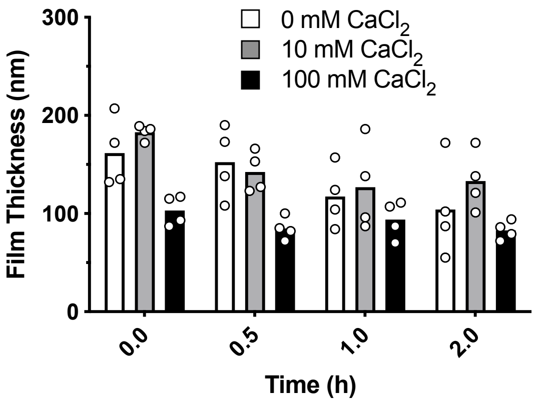

Supplementary Figure 6. **Thicknesses of films after incubation in simulated gastric fluid (SGF) with pepsin.** Films of (polyethylenimine/pectin)$_{10}$ were assembled in 0 mM, 10 mM or 100 mM calcium chloride, then incubated in SGF, pH 1.1 with 1.5 mg/mL (5,000U/mL) pepsin at 37°C, and measured for thickness by profilometry. Symbols represent independent experiments (n=4) and bars represent the mean. Source data are provided as a Source Data file.

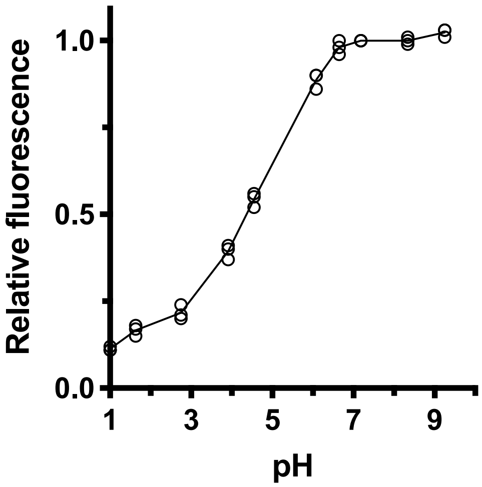

Supplementary Figure 7. **Fluorescence of Oregon Green 488-dextran.** Relative fluorescence of the dye-polymer conjugate as a function of pH. Symbols represent independent experiments (n=3) and the line represents the mean. Source data are provided as a Source Data file.

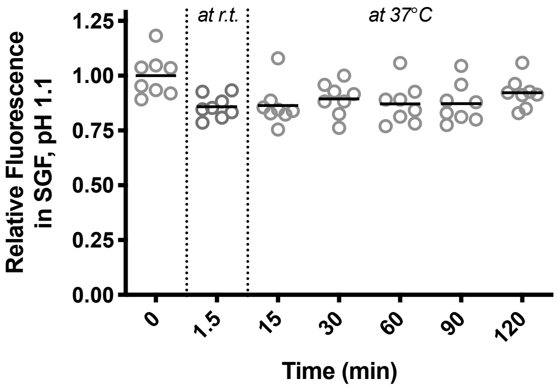

Supplementary Figure 8. **Change in fluorescence after prolonged incubation in simulated gastric fluid (SGF), pH 1.1.** Alginate beads coated with (polyethylenimine/pectin)$_{10.5}$ films were incubated for up to 2 hours at 37°C. The fluorescence from the encapsulated Oregon Green 488-dextran conjugate was measured periodically. Symbols represent individual beads (n=8) and the lines indicate the means. Source data are provided as a Source Data file.

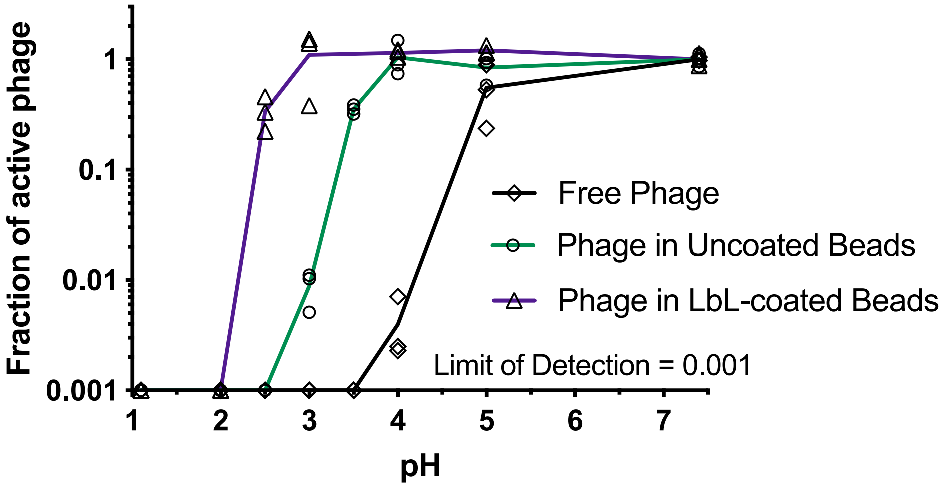

Supplementary Figure 9. **Bacteriophage stability at different pH conditions.** Titration of bacteriophage free in solution, encapsulated in alginate beads, and encapsulated in alginate beads coated with (polyethylenimine/pectin)$_{15.5}$ films incubated in simulated gastric fluid at various pH conditions. Symbols represent independent experiments (n=3) and the lines represent the means. Source data are provided as a Source Data file.

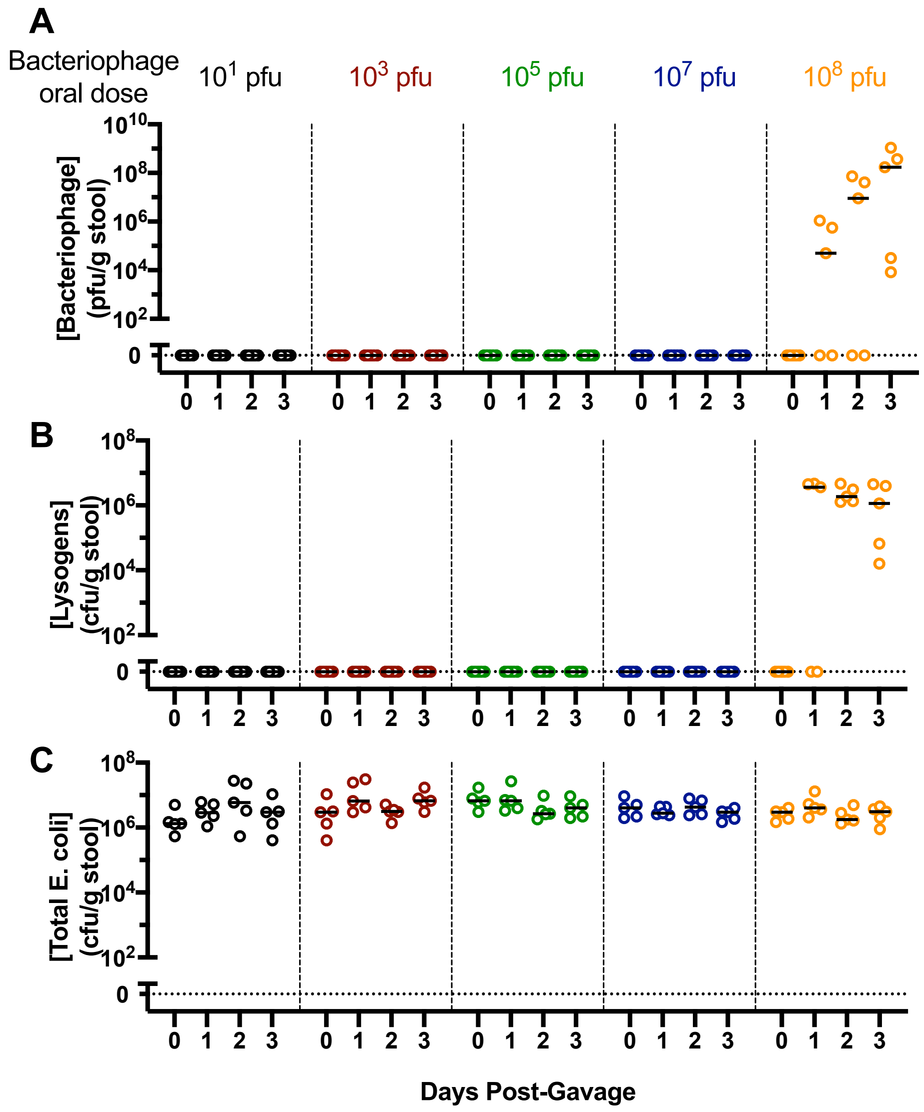

Supplementary Figure 10. **Inactivation of free bacteriophage administered orally to mice.** After free bacteriophage suspended in water was administered by oral gavage to BALB/c mice, the stool was assayed for bacteriophage (A), lysogens (B), and total *E. coli* (C). Symbols represent individual mice (n = 5) and the lines represent the median. Source data are provided as a Source Data file.

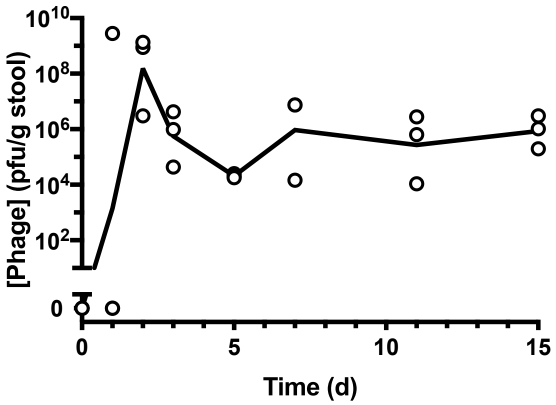

Supplementary Figure 11. **Duration of bacteriophage colonization in vivo.** Bacteriophage encapsulated in $(polyethylenimine/pectin)_{15.5}$ - coated alginate beads were orally administered to mice and show persistent colonization for at least two weeks. Symbols represent individual mice (n = 3) with the line indicating the medians. Source data are provided as a Source Data file.

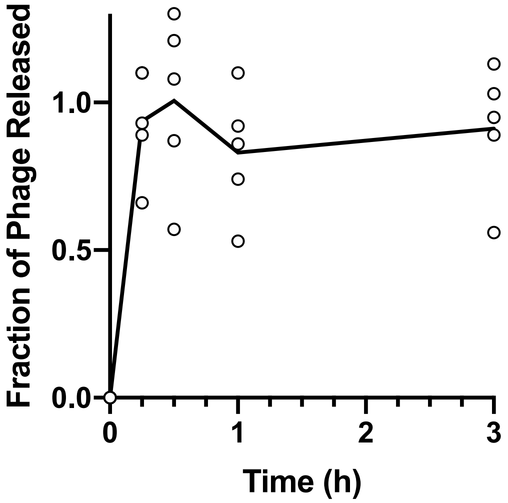

Supplementary Figure 12. ***In vitro* release of phage**. Phage encapsulated in alginate beads coated with (polyethylenimine/pectin)$_{15.5}$ films were incubated at 37°C in phage buffer containing alginate lyase and pectin lyase. Symbols represent independent experiments (n=5) and the line represents the mean. Source data are provided as a Source Data file.

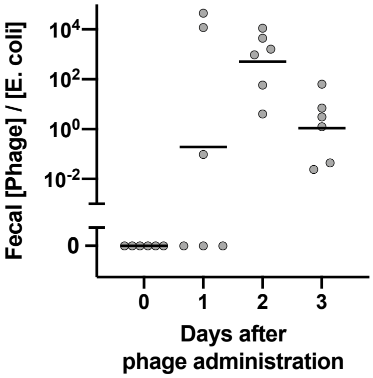

Supplementary Figure 13. **Ratio of phage to bacteria.** The concentration of phage relative to bacteria present in each fecal sample for each mouse. Symbols represent individual mice (n=6) and lines represent the geometric mean. Source data are provided as a Source Data file.

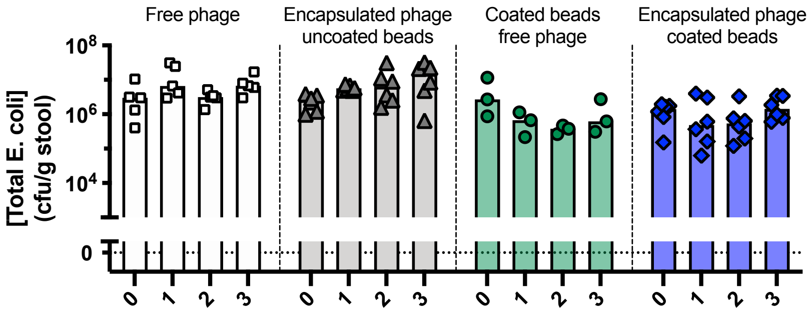

Supplementary Figure 14**. Total fecal *E. coli* in mice.** Mice pre-colonized with *E. coli* were orally administered phage formulations of free phage (n=5), phage encapsulated in uncoated beads (n=6), a mixture of free phage with empty Layer-by-Layer (LbL)-coated beads (n=3), or phage encapsulated in LbL-coated beads (n=6). “LbL-coated” indicates coatings of (polyethylenimine/pectin)$_{15.5}$ films. Symbols represent individual mice with bars indicating the median. Source data are provided as a Source Data file.

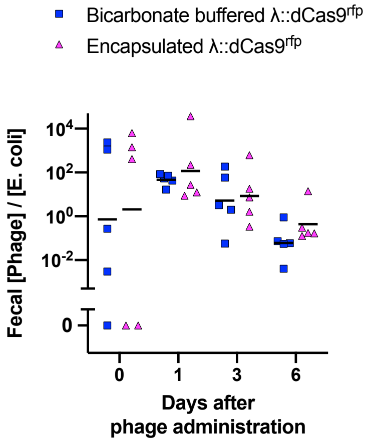

Supplementary Figure 15. **Ratio of phage to bacteria.** The concentration of phage relative to bacteria present in each fecal sample for each mouse. Symbols represent individual mice (n=5 per group) and lines represent the geometric mean. Source data are provided as a Source Data file.

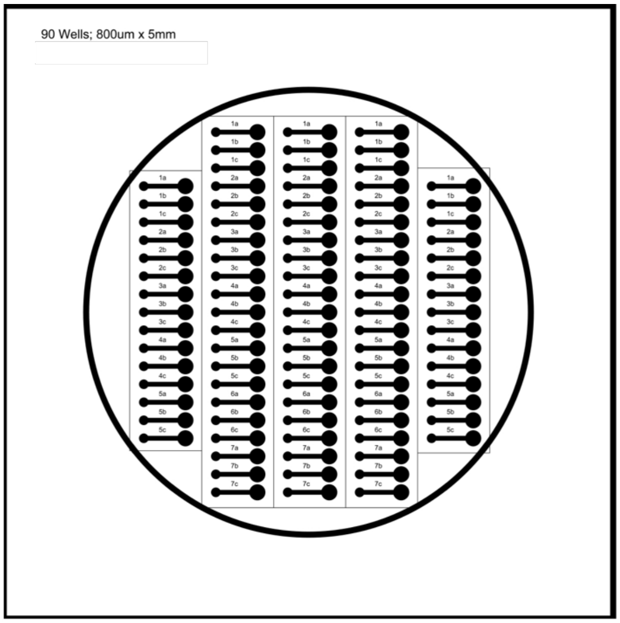

Supplementary Figure 16. **Photolithographic masks used for preparation of master templates.**

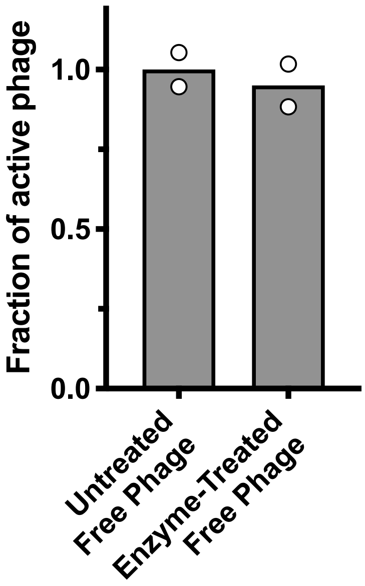

Supplementary Figure 17. **Treatment of bacteriophage with alginate lyase and pectin lyase.** Influence of enzyme incubation on free phage. Symbols represent independent experiments (n=2) and bars represent the means. Source data are provided as a Source Data file.
