# Supplementary material for: In situ reprogramming of gut bacteria by oral delivery
Source: Nat Commun. 2020 Oct 6;11:5030. doi: 10.1038/s41467-020-18614-2 (PMC7538559; doi:10.1038/s41467-020-18614-2)
Supplement: Supplementary file 2 — Reporting Summary [file 41467_2020_18614_MOESM2_ESM.pdf]

nature research

Corresponding author(s): Bryan Hsu

Last updated by author(s): Aug 21, 2020

# Reporting Summary

Nature Research wishes to improve the reproducibility of the work that we publish. This form provides structure for consistency and transparency in reporting. For further information on Nature Research policies, see our Editorial Policies and the Editorial Policy Checklist.

## Statistics

For all statistical analyses, confirm that the following items are present in the figure legend, table legend, main text, or Methods section.

| n/a | Confirmed | |
|---|---|---|
| ☐ | ☒ | The exact sample size ($n$) for each experimental group/condition, given as a discrete number and unit of measurement |
| ☐ | ☒ | A statement on whether measurements were taken from distinct samples or whether the same sample was measured repeatedly |
| ☐ | ☒ | The statistical test(s) used AND whether they are one- or two-sided<br>*Only common tests should be described solely by name; describe more complex techniques in the Methods section.* |
| ☒ | ☐ | A description of all covariates tested |
| ☐ | ☒ | A description of any assumptions or corrections, such as tests of normality and adjustment for multiple comparisons |
| ☐ | ☒ | A full description of the statistical parameters including central tendency (e.g. means) or other basic estimates (e.g. regression coefficient) AND variation (e.g. standard deviation) or associated estimates of uncertainty (e.g. confidence intervals) |
| ☐ | ☒ | For null hypothesis testing, the test statistic (e.g. $F$, $t$, $r$) with confidence intervals, effect sizes, degrees of freedom and $P$ value noted<br>*Give P values as exact values whenever suitable.* |
| ☒ | ☐ | For Bayesian analysis, information on the choice of priors and Markov chain Monte Carlo settings |
| ☒ | ☐ | For hierarchical and complex designs, identification of the appropriate level for tests and full reporting of outcomes |
| ☒ | ☐ | Estimates of effect sizes (e.g. Cohen's $d$, Pearson's $r$), indicating how they were calculated |

*Our web collection on statistics for biologists contains articles on many of the points above.*

## Software and code

Policy information about availability of computer code

| | |
|---|---|
| Data collection | BioTek Gen5 v3.03 was used to collect fluorescence and absorbance plate reader data. Bio-Rad Image Lab v6.0 was used to collect colony fluorescence data. |
| Data analysis | Graphpad Prism v8.4.2 and Microsoft Excel for Mac v16.35 were used for data analysis. Image J v2.0 with FIJI was used to quantify fluorescent intensity. |

For manuscripts utilizing custom algorithms or software that are central to the research but not yet described in published literature, software must be made available to editors and reviewers. We strongly encourage code deposition in a community repository (e.g. GitHub). See the Nature Research guidelines for submitting code & software for further information.

## Data

Policy information about availability of data

All manuscripts must include a data availability statement. This statement should provide the following information, where applicable:

- Accession codes, unique identifiers, or web links for publicly available datasets
- A list of figures that have associated raw data
- A description of any restrictions on data availability

All source data are available in the supplementary information.

# Field-specific reporting

Please select the one below that is the best fit for your research. If you are not sure, read the appropriate sections before making your selection.

☒ Life sciences ☐ Behavioural & social sciences ☐ Ecological, evolutionary & environmental sciences

For a reference copy of the document with all sections, see nature.com/documents/nr-reporting-summary-flat.pdf

# Life sciences study design

All studies must disclose on these points even when the disclosure is negative.

| | |
|---|---|
| Sample size | No statistical methods were used to predetermine sample size. In vivo sample sizes were based on previous publications showing the number of mice necessary to show effect (Weiss et al. Virology (2009); Reyes et al. PNAS (2013)). |
| Data exclusions | No data were excluded. |
| Replication | All in vivo data are from 3-6 individual mice. Experimental replicates are indicated in the figure legends. All replication experiments were successful. |
| Randomization | Age- and sex-matched mice were randomly assigned to experimental groups. |
| Blinding | Handling of animals was not blinded to minimize the possibility of microbial cross-contamination during procedures or by husbandry staff, however samples were blinded during quantification of colony fluorescence and analysis. |

# Reporting for specific materials, systems and methods

We require information from authors about some types of materials, experimental systems and methods used in many studies. Here, indicate whether each material, system or method listed is relevant to your study. If you are not sure if a list item applies to your research, read the appropriate section before selecting a response.

## Materials & experimental systems

| n/a | Involved in the study |
|---|---|
| ☒ | ☐ Antibodies |
| ☒ | ☐ Eukaryotic cell lines |
| ☒ | ☐ Palaeontology and archaeology |
| ☐ | ☒ Animals and other organisms |
| ☒ | ☐ Human research participants |
| ☒ | ☐ Clinical data |
| ☒ | ☐ Dual use research of concern |

## Methods

| n/a | Involved in the study |
|---|---|
| ☒ | ☐ ChIP-seq |
| ☒ | ☐ Flow cytometry |
| ☒ | ☐ MRI-based neuroimaging |

## Animals and other organisms

Policy information about studies involving animals; ARRIVE guidelines recommended for reporting animal research

| | |
|---|---|
| Laboratory animals | Female BALB/c mice, arrived at 6-7 weeks old and acclimated for 1 week |
| Wild animals | No wild animals were used in this study. |
| Field-collected samples | No field samples were collected in this study. |
| Ethics oversight | Animal work was approved by the Harvard Medical School IACUC under protocol number 4966. |

Note that full information on the approval of the study protocol must also be provided in the manuscript.
